# Epigenetic Profiling of *PTPN11* Mutant JMML Hematopoietic Stem and Progenitor Cells Reveals an Aberrant Histone Landscape

**DOI:** 10.3390/cancers15215204

**Published:** 2023-10-29

**Authors:** Roshani Sinha, Mai Dvorak, Ananthakrishnan Ganesan, Larry Kalesinskas, Charlotte M. Niemeyer, Christian Flotho, Kathleen M. Sakamoto, Norman Lacayo, Rachana Vinay Patil, Rhonda Perriman, Alma-Martina Cepika, Yunying Lucy Liu, Alex Kuo, Paul J. Utz, Purvesh Khatri, Alice Bertaina

**Affiliations:** 1 Division of Hematology, Oncology, Stem Cell Transplantation and Regenerative Medicine, Department of Pediatrics, School of Medicine, Stanford University, Stanford, CA 94305, USA; roshani@stanford.edu (R.S.); rpatil1@stanford.edu (R.V.P.); rhondap@stanford.edu (R.P.); acepika@stanford.edu (A.-M.C.); ylliu19@stanford.edu (Y.L.L.); 2 Department of Medicine, School of Medicine, Stanford University, Stanford, CA 94305, USA; maidvora@stanford.edu (M.D.); ananthg@stanford.edu (A.G.); lkalesin@stanford.edu (L.K.); alexjkuo0229@gmail.com (A.K.); pjutz@stanford.edu (P.J.U.); pkhatri@stanford.edu (P.K.); 3 Department of Pediatric Hematology and Oncology, University of Freiburg Medical Centre, 79098 Freiburg im Breisgau, Germany; charlotte.niemeyer@uniklinik-freiburg.de (C.M.N.); christian.flotho@uniklinik-freiburg.de (C.F.); 4 Bass Center for Childhood Cancer and Blood Disorders at Lucile Packard Children’s Hospital, Palo Alto, CA 94304, USA; kmsakamo@stanford.edu (K.M.S.); lacayon@stanford.edu (N.L.)

**Keywords:** *PTPN11* JMML HSPCs, EpiTOF, ATAC-seq, histone modifications, chromatin accessibility

## Abstract

**Simple Summary:**

Juvenile myelomonocytic leukemia (JMML) is a deadly pediatric leukemia with limited treatment options and poor clinical outcomes. Effective targeted treatment strategies are an urgent unmet need. To improve outcomes for this pediatric patient population, we examined the structure of the DNA comprising the genomes of leukemic cells from five JMML patients and compared these to DNA structures from healthy controls. These data allowed us to identify structural features that were unique to the JMML patient DNA. Identification of these JMML-specific changes could guide development of targeted drugs to effectively treat this devastating malignancy. Our work provides a rich resource for additional investigations aimed at identifying and testing strategies designed to treat JMML.

**Abstract:**

Juvenile myelomonocytic leukemia (JMML) is a deadly pediatric leukemia driven by *RAS* pathway mutations, of which >35% are gain-of-function in *PTPN11*. Although DNA hypermethylation portends severe clinical phenotypes, the landscape of histone modifications and chromatin profiles in JMML patient cells have not been explored. Using global mass cytometry, Epigenetic Time of Flight (EpiTOF), we analyzed hematopoietic stem and progenitor cells (HSPCs) from five JMML patients with *PTPN11* mutations. These data revealed statistically significant changes in histone methylation, phosphorylation, and acetylation marks that were unique to JMML HSPCs when compared with healthy controls. Consistent with these data, assay for transposase-accessible chromatin with sequencing (ATAC-seq) analysis revealed significant alterations in chromatin profiles at loci encoding post-translational modification enzymes, strongly suggesting their mis-regulated expression. Collectively, this study reveals histone modification pathways as an additional epigenetic abnormality in JMML patient HSPCs, thereby uncovering a new family of potential druggable targets for the treatment of JMML.

## 1. Introduction

Juvenile myelomonocytic leukemia (JMML) is a deadly pediatric hematologic malignancy characterized by excessive proliferation of monocytes/macrophages, elevated fetal hemoglobin, splenomegaly, and hypersensitivity of leukemia cells to granulocyte–macrophage colony-stimulating factor (GM-CSF) [1,2,3,4,5]. Hematopoietic stem cell transplantation is the only curative option, but post-transplant relapse occurs in ~50% of patients [6,7,8,9]. Developing effective therapeutic strategies has been difficult due to the limited understanding of the disease development. Mutations in *RAS* pathway genes and DNA hypermethylation are major drivers of severe JMML phenotypes [10,11,12,13], the latter confirming the key role of epigenetic changes in disease progression and severity [14,15,16].

Epigenetic changes play an important role in numerous malignant transformations, altering gene expression to promote tumor growth [17,18,19]. In addition to DNA methylation, dysregulation of histone post-translational modifications (HPTM) has been extensively linked to reprogramming in numerous cancer subtypes, including blood cancers such as acute myeloid leukemia (AML), acute lymphoblastic leukemia (ALL), and myelodysplastic syndrome (MDS) [20,21,22]. Histones compress DNA-forming chromatin to regulate eukaryotic gene expression. Histone PTMs are added to, or removed from, the N terminal tails of H2B, H3, and H4 histones by a specific set of enzymes; thus, HPTM pathways are key regulators of chromatin structure and transcriptional profiles [23]. Within hematopoietic cells, the control of histone modifications is key in regulating cell fate [24,25,26,27,28]. However, the landscape of HPTMs in JMML-derived hematopoietic stem and progenitor cells (HSPCs), and their chromatin accessibility profile relative to healthy donor (HD) HSPCs, have not been investigated, and, to date, there have been no published analyses of the HPTM landscape in JMML patients.

Here, we explore the hypothesis that JMML HSPCs carry significant alterations in HPTMs. We have focused this pilot study on JMML patients with gain-of-function mutations in Protein Tyrosine Phosphatase Non-Receptor Type 11 (*PTPN11*; encodes RAS/MAPK signaling pathway protein, SHP-2), which are found in ~38% of all JMML patients and correlate with aggressive disease and hypermethylated DNA [11,12,29]. In addition to JMML, numerous childhood leukemias and solid organ tumors carry gain-of-function *PTPN11* mutations [30,31,32,33]. Further, recent studies have suggested that JMML leukemic initiating cells (LIC) reside within the HSPC compartment [34,35]; thus, we have examined HPTMs within *PTPN11*-mutated JMML HSPCs. Finally, as a hallmark of JMML is splenomegaly, we characterize HSPCs isolated from JMML patient spleens.

We provide the first global comparison of HPTMs and chromatin accessibility in *PTPN11* mutant JMML HSPCs and healthy controls. Using the highly sensitive mass cytometry epigenetic landscape profiling technology Epigenetic Time of Flight (EpiTOF) [36], our data revealed that splenic JMML HSPCs display significant heterogeneity in histone acetylation, phosphorylation, and ubiquitination marks, along with significant reduction in several histone methylation marks when compared to control CD34+ cells from healthy donor umbilical cord blood.

In parallel studies, we used the assay for transposase-accessible chromatin with sequencing (ATAC-seq) to examine the genomic impact of the JMML histone landscape. These data revealed altered chromatin profiles at loci encoding post-translational modification enzymes in JMML HSPCs, strongly suggesting their mis-regulated expression and supporting the reduction in HPTMs identified by EpiTOF. This first such analysis of JMML HSPCs reveals histone modification pathways as an additional epigenetic abnormality in JMML patient HSPCs, thereby identifying a new family of potential druggable targets for the treatment of JMML.

## 2. Materials and Methods

### 2.1. Human Samples

Mononuclear cell samples from JMML patients (*n* = 5) were purified from splenectomy preparations and cryopreserved at the biorepository of the EWOG-MDS, located at Freiburg University Medical Center, Germany. Healthy controls were umbilical cord blood (UCB) from Binns program at Stanford School of Medicine. All human samples were collected from donors with informed consent and compliance with relevant ethical regulations. DNA methylation categories were determined as described previously [13].

### 2.2. Processing Cryopreserved Samples and CD34 Enrichment

Cryopreserved JMML samples were thawed in pre-warmed RPM1 1640 media (Gibco) supplemented with 20% heat-inactivated FBS (ThermoFisher, San Francisco, CA, USA), 1% each of L-glutamine and Penicillin/streptomycin (Gibco, San Francisco, CA, USA). Two sequential DNase1 treatments during thawing allowed optimal recovery of live mononuclear cells (MNCs). MNCs were filtered through 70 µm and CD34+ cells isolated using the CD34 ultrapure microbead kit (Miltenyi Biotec, San Jose, CA, USA).

### 2.3. Epigenetic Landscape Profiling Using Cytometry by Time-of-Flight (EpiTOF)

EpiTOF was as previously described [37,38]. Briefly, Lin-CD34+ cells from JMML and healthy donors were barcoded using the palladium Cell-ID™ 20-Plex Kit (Fluidigm, South San Francisco, CA, USA). All lanthanide-labeled immunophenotypic and intracellular antibodies (Table S1) were prepared using MAXPAR antibody labeling kit (Fluidigm,, South San Francisco, CA, USA). Viability staining was 5 min at RT with 10uM cisplatin (ENZO Life Sciences), then CyTOF Buffer wash (PBS (ThermoFisher, San Francisco, USA), 1% BSA (Sigma-Aldrich, St Louis, MO, USA), 2 mM EDTA (Fisher Scientific, San Francisco, CA, USA), 0.05% sodium azide). Sample pool was sequentially incubated with lanthanide-labeled immunophenotypic antibodies, permeabilized, and labeled with intracellular antibodies [37,38]. Cells were washed and stained with 250 nM 191/193Ir DNA intercalator (Fluidigm, South San Francisco, CA, USA) in PBS, fixed in 1.6% PFA, then washed, filtered (35 μm strainer), and resuspended in ddH2O (ThermoFisher, San Francisco, CA, USA) containing four element calibration beads (Fluidigm, South San Francisco, CA, USA) and analyzed on CyTOF2 (Fluidigm, South San Francisco, CA, USA) in the Stanford Shared FACS Facility. Raw data were concatenated and normalized using calibration beads following manufacturer protocol.

### 2.4. EpiTOF Data Pre-Processing and Analysis and Dimensionality Reduction Analysis

EpiTOF raw data (fcs file) were preprocessed on FlowJo software (FlowJo v9, RRID:SCR_008520, Ashland, OR, USA). Dead cells were removed, and de-barcoding of the palladium-based mass tags performed to select CD34+ cells and export individual sample fcs files.

### 2.5. Data Normalization

We applied a two-step process to normalize EpiTOF data. First, we transformed each measured abundance (HPTM, CPM, total histones) in the raw EpiTOF data to reduce the dynamic range and influence of outliers present in the data:

(1) $${HPTM}_{transformed}=\frac{\sqrt{{HPTM}_{raw}}}{50}$$

We then applied a linear regression for each HPTM using *H*3 and *H*4 as independent variables and using all cells from a single sample for the regression:

(2) $${HPTM}_{i,j}=\beta_{0}+\beta_{1}{H3}_{i}+\beta_{2}{H4}_{i}+\epsilon_{i,j}$$

Here, ${H3}_{i}$ and ${H4}_{i}$ are the transformed abundances of *H*3 and *H*4 in cell *i* and ${HPTM}_{i,j}$ is the transformed abundance of HPTM *j* in cell *i*. We used the residual of the regression, $\epsilon_{i,j}$, as the normalized abundance of HPTM *j* in cell *i*. This was completed to regress out the effect of *H*3 and *H*4 on the HPTM abundance.

### 2.6. UMAP Projection, Clustering of EpiTOF Data, and Cluster Defining HPTMs

We computed UMAP projections [39] on a subset of 40,000 cells (10,000 randomly sampled cells from each panel and disease group) using n_neighbors=15 and min_dist=0.1. Phenograph clustering [40] was then performed on the UMAP space using k=1000.

We then evaluated the cluster-wise median HPTM abundances and sample-wise cell proportions using these 40,000 cells. We compared the sample-wise cell proportions between the healthy subjects (HCB) and patients with JMML (JSP) and used the FDR-adjusted *p*-value computed from Wilcoxon rank-sum test to calculate the significance of the difference in proportions.

To identify cluster-defining, we compared the single-cell resolution abundance of the HPTM in that cluster to the rest of the clusters. We used effect size, computed using Cohen’s D, and FDR-adjusted *p*-value computed from Wilcoxon rank-sum test to calculate the significance of the difference in abundance. The computational methods and code used for EpiTOF data analysis are as previously described [41].

### 2.7. Statistical Analyses

Student’s *t*-test (* *p* < 0.05, ** *p* < 0.01, *** *p* < 0.001, not significant *p* > 0.05) using GraphPad Prism v9 software (RRID:SCR_002798, San Diego, CA, USA).

### 2.8. Omni ATAC Sequencing on Isolated HSPCs

Chromatin accessibility profiling used Omni ATAC sequencing [42]. CD34+ cells from healthy donor UCBs (*n* = 5) and JMML patient spleen samples (*n* = 4) were washed 3x with 1XPBS (without Ca_2+_ and Mg_2+_). Transposition used the Nextera^®^ DNA Sample Preparation Kit (Illumina, San Diego, CA, USA); transposed DNA was purified using MinElute PCR Purification Kit (Qiagen). Preparation/amplification of ATAC libraries is in Corces et al., 2017. Libraries were quantified by qPCR against KAPA Library Quantification kit (KAPA, Roche, San Francisco, CA, USA) and analyzed using Bioanalyzer High-Sensitivity DNA Analysis kit (Agilent); pmol/µL values between 150 bp and 1000 bp were noted for preparing equimolar sample pool for deep sequencing. Deep sequencing was on NextSeq500 (Illumina, San Diego, CA, USA) with 50 million reads/sample of sequencing depth.

### 2.9. Omni ATAC-seq Data Analysis

The de-barcoded individual raw data files were extracted from NextSeq500 as fasta.gz files. Data were analyzed on Partek^®^ Flow^®^ software, v10.0 (Computer software) [43]. Data processing occurred as in [44]. The mitochondrial and ribosomal DNA were filtered out, followed by adapter trimming at <1% per sample. Reads were aligned using Bowtie 2 index against human hg38 assembly on Partek^®^ Flow^®^ software, v10.0 [43]. Reads were filtered to remove duplicates and low mapping quality (<20). Peak calling used MACS2 (BAMPE format) and was annotated against annotation assembly and file hg38 ensemble transcripts release 91 to report genes enriched per peak. TSS enrichment was +5000 bp upstream and downstream of promoters, with minimum mapping quality of 30. For comparison of healthy donor to JMML libraries, reads were quantified and normalized. Prior to annotation, unsupervised hierarchical clustering and dimensionality reduction analysis were performed by PCA. Differentially expressed features were identified with Partek^®^ GSA algorithm to account for varying response of each gene to differing data distributions by applying multiple statistical models. Data were filtered with FDR < 0.05, minimum fold change of +2-folds; and the high or low peak enrichments per gene locus represented as volcano plots, quantified in bar plots and genome tracks shown per sample group (healthy donor UCBs versus JMML spleens).

## 3. Results

### 3.1. A Reduction in Histone Methylation Marks and HMT in Splenic JMML HSPCs

**Figure 1:**
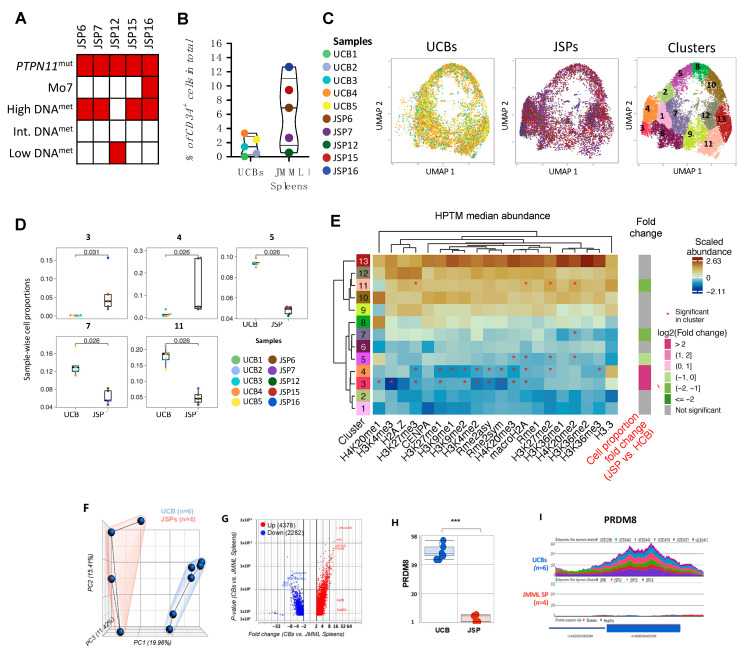
Reduction in histone methylation marks and corresponding HMT in splenic JMML HSPCs. (**A**) Summary of genetic and epigenetic characteristics of EWOG JMML patient samples, including presence of *PTPN11* mutation, Monosomy 7 status, and DNA methylation status (high, intermediate, low). Red = presence; white = absence of characteristics per sample. (**B**) Violin-jitter plots showing total percentage of CD34+ cells isolated from healthy donor (UCB) and JMML (SP) samples. Each patient sample is color-coded as defined in the sample legend. (**C**) Dimensionality reduction analysis was performed on normalized datasets using uniform manifold approximation and projection (UMAP) based on histone methylation post-translational modification (PTM) marks in Table S1. Single-cell level UMAP of UCBs (*n* = 5) or JMML SPs (*n* = 5) are generated, with each dot representing a single cell, and each sample is color-coded as per the legend. Clustering map is also shown, displaying the 13 distinct clusters that were identified with varied distributions of JMML spleens (*n* = 5) and UCBs (*n* = 5) cells within each cluster. (**D**) Each of the 13 clusters had distinct distributions of UCB and JMML cells. Box and whisker plots for clusters with significantly different distributions of JMML and UCB cells are shown. Clusters 5, 7, and 11 have significantly higher number of HSPCs from UCB samples. Clusters 3 and 4 have significantly higher number of JMML HSPCs. Each dot represents a single sample, and each patient sample is color-coded as per the sample legend. No significant differences were observed in cell distributions of JMML versus UCBs in clusters 1, 2, 6, 8, 9, 10, 12, and 13. (**E**) Heatmaps were generated for visualization of median abundance of histone methylation marks per UMAP cluster based on unsupervised hierarchical clustering. Significant abundance or loss of the histone PTM marks that define each cluster distinctly from all the other clusters are marked by an *. The fold change in JMML vs. UCB cell proportion per cluster is also highlighted, with JMML-abundant clusters (3 and 4) being marked magenta and UCB-abundant clusters (5, 7, and 11) marked green, while no significant distinctions between UCB vs. JMML cells proportion were marked gray (1, 2, 6, 8, 9, 10, 12, and 13). Each cluster is defined by distinct histone PTMs signature. Overall differences in histones PTM marks profiles between clusters as well as between individual histone PTMs are denoted with dendrograms. (**F**) Clustering of bulk ATAC sequencing (Omni-ATAC protocol [42]) on CD34+ cells from JMML spleens (*n* = 4: JSP6, JSP12, JSP15, and JSP16) and healthy donor controls (UCBs; *n* = 6) shows the two groups are distinct by dimensionality reduction analysis (PCA). Red outlined = JMML spleen samples, blue = heathy UCBs. (**G**) Volcano plot of GSA on normalized reads per UCB and JMML spleen samples; fold change on *x*-axis, *p*-value on *y*-axis. Each dot is a chromosome region; peaks are reads at specific regions per sample. Labeled genes in the volcano plot represent regions at genes of interest that have significantly differential peak enrichments/read counts between controls and JMML spleens. Further, 4378 regions showed higher peak enrichments in UCBs (i.e., down in JMML), while 2282 regions had increased peak enrichments in JMML (up in JMML). (**H**) Bar plot of reads at loci encoding the histone-modifying enzyme PRDM8, a histone methyltransferase, in control UCB vs. JMML spleen samples and (**I**) corresponding gene tracks representative of peaks/read counts per sample show absence of peaks at the PRDM8 locus in JMML. Multiple *t*-tests were performed for statistical analyses (*** *p* < 0.001). HSPC: hematopoietic stem or progenitor cells; UCB: umbilical cord blood; SP: spleen; H-SNE: heuristical–stochastic neighbor embedding; PTM: post-translational modifications; UMAP: uniform manifold approximation and projection.

DNA hypermethylation is a prognostic feature of JMML, and the DNA demethylating agent azacytidine has shown promise (NCT02447666) [36], but there remains an urgent need for more effective targeted treatment strategies for JMML [7]. To understand JMML development within the stem cell compartment and to identify new therapeutic targets, we characterized histone modifications and chromatin accessibility in the CD34+ cells of five *PTPN11*-mutated JMML spleen-derived HSPCs (Figure 1A). We hypothesized that HPTMs are dysregulated in JMML-patient-derived HSPCs. Therefore, we used EpiTOF [37,38] to investigate HPTMs in the HSPCs of the five primary *PTPN11*-mutated JMML spleen samples [36]. We focused this initial study on the most prevalent and aggressive subtype of JMML (*PTPN11-*mutated, found in >35% of JMML patients).

**Figure 2:**
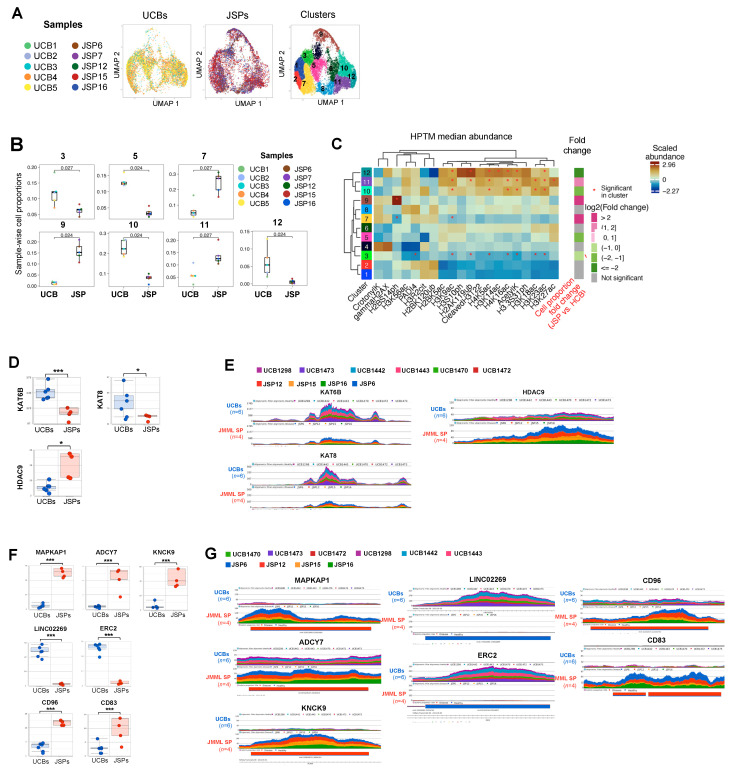
Loss of histone acetylation and phosphorylation markers and corresponding HATs in primary JMML splenic HSPCs. EpiTOF performed on HSPCs (CD34+ cells) from healthy donor controls (UCB *n* = 5) and JMML patient samples (SP *n* = 5) at the single-cell level. (**A**) Dimensionality reduction analysis was performed on normalized datasets using uniform manifold approximation and projection (UMAP) based on histone acetylation, phosphorylation, and ubiquitination post-translational modification (PTM) marks in Table S1. Single-cell level UMAP of UCBs (*n* = 5) or JMML SPs (*n* = 5) are generated, with each dot representing a single cell, and each sample is color-coded as per the legend. Clustering map is also shown, displaying the 12 distinct clusters that were identified with varied distributions of JMML spleens (*n* = 5) and UCBs (*n* = 5) cells within each cluster. (**B**) Each of the 12 UMAP clusters had distinct distributions of UCB and JMML cells. Box and whisker plots for the clusters with significantly different distributions of JMML vs. UCB cells are shown. Clusters 3, 5, 10, and 12 have significantly higher number of HSPCs from UCB samples, while clusters 7, 9, and 11 have significantly higher number of JMML HSPCs. Each dot represents a single sample, and each sample is color-coded as per the legend. No significant differences in cell distribution between JMML versus UCBs were observed in clusters 1, 2, 4, 6, and 8. (**C**) Heatmaps were generated for visualization of median histone PTMs abundance per UMAP cluster based on unsupervised hierarchical clustering. Significant abundance or loss of the histone PTM marks that define each cluster distinctly from all the other clusters are marked by an *. The fold change in JMML vs. UCB cells proportion in each cluster is also highlighted, with JMML-abundant clusters (7, 9, and 11) being marked in magenta and UCB-abundant clusters (3, 5, 10, and 12) marked in green, while no significant distinctions between UCB vs. JMML cells proportion are marked gray (1, 2, 4, 6, and 8). Each cluster is defined by a distinct trend of histone PTMs signatures. The overall differences in histones PTM marks profiles between clusters as well as between individual histone PTMs are denoted with dendrograms. (**D**) Bulk ATAC-seq was performed on CD34+ cells from JMML spleens (*n* = 4: JSP6, JSP12, JSP15, and JSP16) and healthy donor controls (UCBs; *n* = 6). Bar plots of reads at loci encoding histone-modifying enzymes Kat6B, Kat8, HDAC5, and HDAC9 in control UCB vs. JMML spleen samples and (**E**) corresponding gene tracks representative of peaks/read counts per sample. (**F**) The most significantly distinct loci between UCBs and JMML SPs summarized with LS mean read counts per sample. Bar plots high read counts in JMML include MAPK-AP1, ADCY7, KNCK9, CD96, CD83, and CD200. Low read counts in JMML at loci encoding LINC02269 and ERC2. Bar plots of reads at these loci along with their respective (**G**) gene tracks in JMML spleens compared to UCB controls are shown. Multiple *t*-tests were performed for statistical analyses (* *p* < 0.05, *** *p* < 0.001). Each sample was color-coded consistently in the entire Figure 1, as shown in the legends of Figure 1B,C. HSPC: hematopoietic stem or progenitor cells; HD: healthy donor; UCB: umbilical cord blood; SP: spleen; UMAP: uniform manifold approximation and projection.

JMML patients present with splenomegaly, for which they often undergo splenectomy; thus, the spleen provides a rich source of leukemia cells, as evident from the high number of CD34+ cells we recovered from most JMML patients (2–12% in JMML spleens compared to 0.3–3.5% in healthy donor umbilical cord blood (HD UCBs) (Figure 1B). The leukemogenic capacity of JMML splenic CD34+ cells has been previously confirmed in humanized mouse models through serial transplantation [45,46]. Further, as healthy pediatric spleen tissue is unavailable, and comparable adult spleens contain <0.1% CD34+ cells [47,48], we used HD UCBs as controls for primary JMML spleens, consistent with previous studies [35,46]. We investigated histone methylation (Figure 1), acetylation, ubiquitination, and phosphorylation PTMs (Figure 2) in the CD34+ compartment from HSPCs of five JMML patient (pt) samples alongside five HD UCB controls.

To compare the distributions of the histone methylation marks in isolated CD34+ cells from JMML vs. HD UCBs, we first performed dimensionality reduction analyses using uniform manifold approximation and projection (UMAPs; [39]) and then clustered the cells using PhenoGraph [40] (Figure 1C). These analyses were performed as previously described [41]. The histone methylation profiles grouped the CD34+ cells from JMML and HD UCB controls into 13 distinct clusters (Figure 1C; right panel).

Clusters 3 and 4 had significantly higher proportions of JMML-derived HSPCs than HD UCBs, whereas clusters 5, 7, and 11 had significantly lower proportions of JMML HSPCs (Figure 1D). The remaining clusters (1, 2, 6, 8, 9, 10, 12, and 13) showed no significant differences in cell proportions. Overall, comparative analysis of the median abundance of each individual methylation mark in clusters 3, 4, 5, 7, and 11 revealed that several were significantly different between JMML HSPCs and HD UCBs and when compared to all other clusters combined (Figure 1E). This analysis was not performed for clusters 1, 2, 4, 6, and 8 as there was no significant difference in JMML vs. HD UCB cell proportions. In Figure 1E, the heatmap shows the median abundance of individual HPTM marks at the single-cell level in each cluster. Additionally, the fold-change between JMML and HD UCB HSPC proportions per cluster is shown to the right.

We focused our attention on methylation marks that were significantly different in clusters enriched for JMML HSPCs (clusters 3 and 4) or clusters significantly enriched for HD UCB HSPCs (clusters 5, 7, and 11), reasoning that these would reveal disease-specific features.

Several methylation marks were uniquely lower in clusters 3 and 4, which were enriched for JMML HSPCs (Figure 1D,E-Fold change). Specifically, we observed significantly lower abundance of H3K27me3, H3K27me1, H3K9me2, H3K4me2, Rme2asy, and Rme2sym when compared to all other clusters (Figure 1E, Figures S1 and S2). The UMAP cluster 3 also had significantly lower H4K20me1 and H3K4me3, whereas cluster 4 had significantly lower H3K9me1 and H3K36me3 (Figure 1E, Figures S1 and S2). These data suggest that these specific methylation marks are uniquely and significantly reduced in JMML HSPCs.

In contrast, clusters 5 and 7, which were significantly enriched for HD UCB HSPCs, showed a specific and significant reduction in median abundance of the histone methylation mark H4K20me2 (Figure 1E, Figures S1 and S2). Curiously, however, cluster 11, which was also enriched for HD UCB HSPCs, showed a significantly higher median abundance of H4K20me2 (Figure 1E, Figures S1 and S2). We note that, overall, cluster 11 has higher median abundance of several methylation marks. For example, while the JMML-enriched clusters 3 and 4 were significantly lower for H3K27me3, the HD-UCB-HSPC-enriched cluster 11 was significantly higher for this methylation mark (see column 4, Figure 1E).

To identify whether the histone hypomethylation patterns that we observed in the overall JSPs vs. HD UCBs might be driven by specific HSPC subsets, we performed dimensionality reduction analysis on isolated HSPC subsets (see Supplementary Table S3) from each JMML HSPC and HD UCB sample. Phenotypically, we observed considerable differences in the distribution of distinct HSPC subsets in JMML vs. HD UCBs (Figure S3) with significantly higher hematopoietic stem cells (HSCs), CD34+ CD38− CD45RA+ CD90+ (Leukemic-MPP, Figure S3B), and CD34+ CD38+ CD45RA+ CD90+ (Figure S3C) subsets. The latter two subsets were found almost exclusively in JMML samples compared to HD UCBs (Figure S3B,C). Although not statistically significant, the JMML samples also had a higher percentage of granulocyte–monocyte progenitors (GMPs) and lower lymphoid–myeloid primed progenitors (LMPPs) when compared to HD UCBs (Figure S3B,C). However, we found heterogenous clustering of the HSPC subsets from both JMML and HD UCBs in the UMAP space generated based on the histone methylation panel (Figure S4), making it difficult to identify any unique HPTM signatures that were specific to a JMML or HD HSPC subset.

Our data show that specific methylation marks are reduced in JMML HSPCs, suggesting alterations in the chromatin structure. Thus, we next analyzed chromatin accessibility profiles using bulk ATAC-seq on CD34+ cells from JMML (*n* = 4; pts #6, #12, #15, and #16) and HD UCB (*n* = 6) samples [42]. Dimensionality reduction using principal component analysis revealed distinct clustering of healthy versus JMML samples, indicative of differential chromatin accessibility in these two groups (Figure 1F). The heterogeneity within disease samples (red outline, Figure 1F) likely reflects the diversity in patient characteristics, including disease stage at diagnosis, age, gender, and outcome (Supplementary Table S2). Differential accessibility analysis identified 4378 loci with significantly decreased and 2282 loci with significantly increased chromatin accessibility in JMML vs. UCBs (Figure 1G). These data confirm that there are significant global changes to chromatin structure in JMML vs. HD UCB CD34+ cells.

We specifically examined loci for genes encoding histone methyltransferases (HMT) and demethylases (DNMT). Figure 1H and 1I show a comparison of the chromatin accessibility profiles of JMML vs. UCB HSPCs at the *PRDM8* locus. These data revealed a complete loss of peaks at the PRDM8 locus that was unique to the JMML HSPCs (Figure 1H,I). PRDM8 catalyzes H3K9me [49], and our observation of the absence of peaks at the PRDM8 locus in all JMML samples is consistent with the significant reduction in H3K9me1 and H3K9me2 methylation we observed in JMML-HSPC-enriched clusters 3 and 4 (columns 7 and 8, Figure 1E). We conclude that the reduction in H3K9me marks observed in JMML-enriched clusters 3 and 4 is likely due to a loss of PRDM8 expression.

### 3.2. Heterogenous Histone Acetylation, Ubiquitination, and Phosphorylation Profiles in Primary JMML Splenic HSPCs

Next, we compared the histone acetylation, phosphorylation, and ubiquitination marks in JMML HSPCs (*n* = 5) to control HD UCB HSPCs (*n* = 5) by again performing dimensionality reduction with UMAPs followed by clustering with PhenoGraph (Figure 2), as previously described [41]. Histone acetylation, phosphorylation, and ubiquitination data grouped the CD34+ cells from JMML HSPCs and HD UCB controls into 12 distinct clusters (Figure 2A). We observed that the JMML HSPCs were significantly enriched in clusters 7, 9, and 11. In contrast, clusters 3, 5, 10, and 12 were enriched for HD UCB HSPCs with significantly lower proportions of JMML HSPCs (Figure 2B).

As for our analysis of the methylation marks in Figure 1, we analyzed which HPTMs were uniquely affected in JMML-enriched or HD-UCB-enriched clusters, and whether their median abundance was significantly up- or downregulated relative to all other clusters (Figure 2C). We found that, while JMML-enriched clusters 7 and 9 had significantly less H2BS14ph, JMML-enriched cluster 9 was significantly increased at this phosphorylation site, indicating strong heterogeneity across patients at this site. Cluster 11 showed significantly more HPTM at H3K27ac. Although trends were apparent, we observed no other statistically significant changes in HPTM marks that were uniquely affected in JMML-enriched HSPCs.

We then evaluated HPTM signatures unique to HD UCB HSPC clusters 3, 5, 10, and 12 (Figure 2B,C-Fold change) and found only two changes were unique to the HD UCB cells. In cluster 3, we observed significantly reduced levels of PAD14 (Figure 2C, Figures S5 and S6), while cluster 12 had a significantly higher proportion of H3K14ac (Figure 2C, Figures S5 and S6).

To determine whether the heterogenous histone acetylation, phosphorylation, and ubiquitination patterns that we observed in JMML HSPCs vs. HD UCBs might be driven by specific HSPC subsets, we performed dimensionality reduction analysis on isolated HSPC subsets (Table S3) from each JSP and UCB sample. As for the histone methylation panel (Figure S4), we found heterogenous clustering of all the HSPC subsets from JSPs and UCBs in the UMAP space generated based on the histone acetylation, phosphorylation, and ubiquitination panel (Figure S7). We identified 14 distinct clusters in the UMAP space (Figure S7A); however, each cluster consisted of some proportion of every HSPC subset from UCBs as well as JSPs (Figure S7B,C). Thus, we could not assign the unique HPTM signatures to any specific disease or healthy HSPC subsets.

We next used ATAC-seq to examine gene loci encoding HPTM enzymes that catalyze histone acetylation, phosphorylation, and ubiquitination marks, as we did for the methylating enzymes. These analyses revealed that HD UCB HSPCs had significant peak enrichments, at histone acetyltransferases (HATs) Kat6b (catalyzes acetylation of H3K23 [50,51]), and Kat8 (catalyzes H4K16ac [52]), when compared to JMML samples (Figure 2D,E). These significantly higher open chromatin regions at these HATs loci in UCB HSPCs were consistent with the significantly higher abundance of H3K23ac and H4K16ac in all three UCB-HSPC-enriched clusters (Figure 2C). Interestingly, the JMML-enriched cluster 11 also demonstrated an upregulation of these acetylation marks. Overall, however, these data suggest reduced Kat6b and Kat8 HAT activities in JMML HPSCs, leading to a reduction in HPTM acetylation. Consistent with this conclusion, we also observed significant peak enrichments at histone deacetylases HDAC9 and HDAC5 in the JMML samples (Figure 2D,E). Collectively, we conclude that there is reduced histone acetylation in the majority of JMML HSPCs when compared to HD UCBs (Figure 2).

We also identified other loci for which the ATAC-seq data showed enriched peaks specifically in JMML HSPCs. Genes at these loci included mitogen-activated protein-kinase-associated protein 1 (MAPK-AP1), a component of the mTOR2 pathway [53], potassium channel gene KCNK9, and adenylate cyclase 7 (ADCY7) (Figure 2F,G). Both KCNK9 and ADCY7 are often found overexpressed in human cancers [54,55,56]. In contrast, peaks at the neurotransmitter-release-associated gene ERC2 and long intergenic non-coding RNA 2269 (LINC02269) were significantly reduced in the JMML HSPCs (Figure 2F,G). JMML-HSPC-specific significant peak enrichments were also observed at immunophenotypic markers CD96, CD83, and CD200, although there was heterogeneity between patient samples at CD83 and CD200 (Figure 2F,G). These data are in agreement with Louka et al.’s finding of CD96 expression on putative JMML LICs [35].

## 4. Discussion

The largest hurdle in treating JMML patients is the lack of druggable targets [7]. As reversible features, epigenetic changes have great potential for therapeutic intervention. However, while DNA hypermethylation is a prognostic feature of aggressive disease, and the DNA demethylating agent, 5-azacitidine, has shown promise (NCT02447666) [36], there is an urgent need for targeted, more effective treatment strategies to cure JMML. To understand JMML development within the stem cell compartment and to identify new therapeutic targets, we have characterized the histone modifications and chromatin accessibility in the CD34+ compartment of five *PTPN11*-mutated JMML spleen-derived HSPCs. To the best of our knowledge, these data represent the first such analyses.

We found that the spleen-derived *PTPN11*-mutated JMML HSPCs exhibited significantly reduced histone methylation marks that are unique to JMML HSPC at H3K27me1-3, H3K9me1-2, H3K4me2, Rme2asy, Rme2sym, H4K20me1, and H3K36me3. Indeed, JMML-enriched UMAP clusters 3 and 4 had overall significantly reduced histone methylation marks when compared to all the other clusters, suggesting a consistent histone hypomethylation signature in JMML samples. We also found significant, although heterogeneous, changes at H2BS14ph, whereby one JMML-enriched cluster contained cells that were significantly increased for phosphorylation marks at this site, while another JMML-enriched cluster was significantly reduced. Additional JMML-HSPC-specific changes were observed at H3K27ac, which was significantly increased in a cluster enriched for JMML cells. We similarly identified HD-UCB-HSPC-specific HPTMs and observed (i) a significant reduction in the median abundance of the histone methylation mark H4K20me2 in two out of the three clusters (the third HD-UCB-enriched cluster showed an increase at this site), and (ii) significantly reduced levels of PAD14 and a significantly higher proportion of H3K14ac. Taken together, these data demonstrate significant differences in HPTMs between the two sample groups.

Loss of HPTM marks such as those observed in our analysis of JMML HSPCs are signature events in numerous malignancies, including H3K23ac in solid tumor models such as breast cancer, KRAS-mutated colorectal cancer, and non-small-cell lung cancer [51,57]; H3S10ph in aggressive solid tumors, including colon cancer [58,59]; and H3K36me2, H3K36me2, and H3K27me2 in leukemic transformation in AML [60] and poor AML patient prognosis [61,62]. Consistent with this loss in JMML, our ATAC-seq analysis showed decreased peak enrichments at histone acetyltransferases *Kat6b* and *Kat8* and methyltransferase *PRDM8* but increased peak enrichments at histone deacetylases *HDAC9* and *HDAC5*. *Kat6b* and *Kat8* are specific for H4K16ac and H3K23ac, respectively. The concurrent loss of *Kat8* and H4K16ac has been shown in aberrant gene expression in AML [63,64], suggestive of a similar mechanism in JMML. Similarly, *Kat6b* plays a tumor suppressor role in non-small-cell lung cancer wherein its loss leads to cancer growth [65]. *PRDM8* is also a strong tumor suppressor that is downregulated in hepatocellular carcinoma [64,66]; thus, loss of peaks at this locus supports an analogous role in JMML onset. Indeed, a recent study showed that knockout of *PRDM8* in iPSCs led to impaired hematopoietic differentiation and myeloid-biased malignant transformation [66].

Increases in HDAC activity, as our ATAC-seq data suggest for HDAC5 and HDAC9 in JMML spleen HSPCs, have been identified in a variety of tumor types. Several class I HDAC inhibitors are approved for cancer treatment (i.e., vorinostat, romidepsin, and belinostat). However, these drugs do not inhibit class II HDACs, such as HDAC5 and HDAC9 [67]. Instead, the pan-HDAC inhibitor, panobinostat, which has shown promise treating patients with multiple myeloma [68], has also demonstrated targeted killing of primary JMML HSPCs, interestingly showing greater efficacy than 5-azacytidine in vitro [69]. We also note that TMP195, a first-in-class highly selective class II HDAC inhibitor, has shown potent activity in breast cancer mouse model studies [70], making it an attractive candidate to test in JMML patients who display increased HDAC5 and HDAC9 activity.

We also observed reduced peaks at numerous other loci. Indeed, overall, we observed largely closed, heterochromatic regions in JMML HSPCs, suggesting significant repression of gene expression. However, specific loci such as *MAPKAP1* displayed peak enrichments. *MAPKAP1/Sin1* is linked to *RAS* and *mTOR2* pathways and is known to drive *Akt* phosphorylation [71,72]. *RAS* pathway mutations, including elevation of *pAKT*, drive JMML, and preclinical evidence suggests a therapeutic role for mTOR inhibition in JMML [2,72,73]. Thus, our data suggest a possible link between *RAS* and *mTOR2* pathways in JMML via *MAPKAP1*, making this pathway a putative therapeutic target. We also found highly enriched peaks at the potassium channel gene *KCNK9* in all JMML samples. Others have shown that overexpression of this gene promotes malignancy in several cancers [56]. In addition, several immunophenotypic gene regions were differentially regulated in JMML. *CD83* is a marker of AML LICs, and therapeutic targeting using *CD83*-CAR T cells has shown preliminary success in humanized mouse models of AML [74], suggesting it could be a potential therapeutic target in JMML. Interestingly, another known marker of AML LICs, *CD200*, had significantly higher read counts in JMML HSPCs when compared to UCBs. *CD200* is upregulated in the most resistant pediatric AMLs and confers immunosuppressive properties when co-cultured with engineered T regulatory 1 cells [75,76]. 

Collectively, our data identify aberrant histone PTMs in splenic JMML HSPCs from patients carrying *PTPN11* gain-of-function mutations and implicate the activity of PTM-modifying enzymes as responsible for the observed changes. We are currently exploring these changes, including confirming transcriptional profiles using RNAseq and isolating specific HSPC cell subsets to identify specific cell lineages in which these changes occur.

## 5. Conclusions

In conclusion, we have identified aberrant histone PTMs in splenic *PTPN11*-mutated JMML HSPCs and revealed differential accessibilities at the loci of the respective HPTM-modifying enzymes. Additionally, using ATAC-seq, we have shown significant heterochromatin (closed) at key genomic regions that encode for known tumor suppressors, HATs, and HMTs, suggesting their loss of expression. The newly identified signatures of aberrant histone PTMs, such as the targeted histone hypoacetylation and hypomethylation, may be used in the future as prognostic or predictive epigenetic markers in patients with JMML caused by *PTPN11* mutation. Most importantly, we have identified surface markers such as CD96, CD83, and CD200 as potential targets for cell therapy.

The data obtained in this pilot analysis will guide future work analyzing histone PTM profiles found in HSPCs from other JMML subtypes, i.e., those carrying causative mutations in the *NF1*, *KRAS*, *NRAS,* or *CBL* genes. Together with *PTPN11*, ~90% of patients harbor mutation in one of these five genes. Comparison of PTM profiles across these subtypes will likely reveal pan-leukemic as well as specific changes that drive JMML subtype dysregulation. Furthermore, we are currently validating the HPTM changes in the *PTPN11*-mutated JMML patient samples from this study, including identifying the transcriptional profiles using single-cell RNAseq to clarify if these changes occur in specific cell lineage(s). As our studies progress to other JMML subtypes, we will incorporate comparable analyses to develop an in-depth understanding of the landscape of histone modifications and chromatin profiles across JMML patients. Overall, our studies will inform common and distinct underlying features that highlight the rich biology governed by PTMs in JMML, thus revealing potential new therapeutic avenues.

## Data Availability

The raw data files that support the findings of this study will be made openly available on code ocean.

## Supplementary Materials

The following supporting information can be downloaded at: https://www.mdpi.com/article/10.3390/cancers15215204/s1, Figure S1: Key histone methylation marks that defined clusters with significantly distinct JMML vs UCB HSPC proportions: Dimensionality reduction analysis was performed on normalized datasets using uniform manifold approximation and projection (UMAP) based on histone methylation post translational modification (PTM) marks. Figure S2: Distinct histone methylation signatures in JMML versus UCB HSPCs using UMAP clustering. Figure S3: HSPCs identified in JMML samples compared to healthy donor controls. Figure S4: Reduction in histone methylation marks in splenic JMML HSPC subsets. Figure S5: Key histone acetylation, phosphorylation, and ubiquitination marks that defined clusters with significantly distinct JMML vs UCB HSPC proportions: Dimensionality reduction analysis was performed on normalized datasets using uniform manifold approximation and projection (UMAP) based on histone acetylation, phosphorylation, and ubiquitination post translational modification (PTM) marks. Figure S6: Distinct histone acetylation, phosphorylation, and ubiquitination signatures in JMML versus UCB HSPCs using UMAP clustering. Figure S7: Heterogenous histone acetylation, ubiquitination and phosphorylation markers in primary JMML splenic-HSPC subsets. Table S1. EpiTOF panels of lanthanide-labeled immunophenotypic and intracellular antibodies. Table S2. Clinical and genetic characteristics of JMML patients cohort. Table S3. Phenotype of hematopoietic stem and progenitor subsets identified in UCBs and. JMML patients samples.
