# Supplementary material for: Epigenetic Profiling of PTPN11 Mutant JMML Hematopoietic Stem and Progenitor Cells Reveals an Aberrant Histone Landscape
Source: Cancers (Basel). 2023 Oct 29;15(21):5204. doi: 10.3390/cancers15215204 (PMC10650722; doi:10.3390/cancers15215204)
Supplement: Supplementary file 1 [file cancers-15-05204-s001.zip › cancers-2673483-supplementary.pdf]

Supplementary Material: Epigenetic Profiling of *PTPN11* Mutant JMML Hematopoietic Stem and Progenitor Cells Reveals an Aberrant Histone Landscape

Table S1. EpiTOF panels of lanthanide-labeled immunophenotypic and intracellular antibodies.

Panel 1						Panel 2				
**Metal**	**Marker**	**Manufacturer**	**Type**	**Clone**		**Metal**	**Marker**	**Manufacturer**	**Type**	**Clone**
89Y	CD45	Fluidigm	Mouse IgG1	HI30		89Y	CD45	Fluidigm	Mouse IgG1	HI30
141Pr	H3	CST	Rabbit mAb	D1H2		141Pr	H3	CST	Rabbit mAb	D1H2
142Nd	γ-H2AX	CST	Rabbit mAb	20E3		142Nd	Arg-me1	Abcam	Mouse IgG1	5D1
143Nd	H2BK5ac	CST	Rabbit mAb	D5H1S		143Nd	Arg-me2 (sym)	CST	Rabbit mAb	13222
144Nd	H3S10ph	Active Motif	Mouse IgG1	MABI 0312		144Nd	H3K4me2	Active Motif	Mouse IgG1	MABI 0303
145Nd	CD14	BioLegend	Mouse IgG2a	M5E2		145Nd	CD14	BioLegend	Mouse IgG2a	M5E2
146Nd	CD33	BioLegend	Mouse IgG1	WM53		146Nd	CD33	BioLegend	Mouse IgG1	WM53
147Sm	H4K5ac	Active Motif	Mouse IgG1	MABI 0405		147Sm	H3K9me2	Biolegend	Mouse IgG1	5E5-G5
148Nd	CD34	BD	Mouse IgG1	8G12		148Nd	CD34	BD	Mouse IgG1	8G12
149Sm	Cleaved H3 (Thr22)	CST	Rabbit mAb	D7J2K		149Sm	H3K9me1	Biolegend	Mouse IgG1	7E7.H12
150Nd	H3.3S31ph	Active Motif	Mouse IgG2b	1A8G10		150Nd	H3K36me3	RevMab	Rabbit mAb	RM155
151Eu	H3K23ac	RevMab	Rabbit mAb	RM169		151Eu	H3K27me1	Active Motif	Mouse IgG2a	MABI 0321
152Sm	H3K9ac	Active Motif	Mouse IgG2a	2G1F9		152Sm	Arg-me2 (asy)	CST	Rabbit mAb	13522
153Eu	H2BS14ph	CST	Rabbit mAb	D67H2		153Eu	H3K36me2	Active Motif	Mouse IgG1	MABI 0332
154Sm	H2AK119ub	CST	Rabbit mAb	D27C4		154Sm	H3K27me2	Active Motif	Mouse IgG2a	MABI 0324
155Gd	CD45RA	BioLegend	Mouse IgG2b	HI100		155Gd	CD45RA	BioLegend	Mouse IgG2b	HI100
156Gd	H3K18ac	RevMAb	Rabbit mAb	RM166		156Gd	H4K20me2	Active Motif	Mouse IgG1	MABI 0422
158Gd	H3K56ac	Active Motif	Mouse IgG1	12.1		158Gd	H3.3	Abcam	Rabbit mAb	EPR17899
159Tb	CD90	STEMCELL	Mouse IgG1	5E10		159Tb	CD90	STEMCELL	Mouse IgG1	5E10
160Gd	PADI4	OriGene	IgG2a	OTI4H5		160Gd	H4K20me3	BioLegend	Mouse IgG1	6F8-D9
161Dy	H2BK120ub	CST	Rabbit mAb	D11		161Dy	Macro-H2A	Millipore	Mouse IgG2b	14G7
162Dy	Crotonyl-Lys	PTM Biolabs	Mouse IgG	4D5		162Dy	H3K4me3	Life	Rabbit IgG	G.532.8
163Dy	H3R2cit	Abcam	Rabbit mAb	EPR17703		163Dy	H2A.Z	Abcam	Rabbit mAb	[EPR6171(2)(B)]
164Dy	H3K14ac	CST	Rabbit mAb	D4B9		164Dy	H3K36me1	Abcam	Rabbit mAb	EPR16993
165Ho	Acetyl-lys	RevMab	Rabbit IgG	RM101		165Ho	H3K27me3	Active Motif	Mouse IgG1	MABI 0323
166Er	CD10	BioLegend	Mouse IgG1	HI10a		166Er	CD10	BioLegend	Mouse IgG1	HI10a
167Er	CD11b	BioLegend	Mouse IgG1	ICRF44		167Er	CD11b	BioLegend	Mouse IgG1	ICRF44
168Er	H4K16ac	CST	Rabbit mAb	E2B8W		168Er	H4K20me1	Active Motif	Mouse IgG	5E10-D8
169Tm	CD123	BD	Mouse IgG1	9F5		169Tm	CD123	BD	Mouse IgG1	9F5
170Er	CD3	BioLegend	Mouse IgG1	UCHT1		170Er	CD3	BioLegend	Mouse IgG1	UCHT1
171Yb	CD38	BioLegend	Mouse IgG1	HIT2		171Yb	CD38	BioLegend	Mouse IgG1	HIT2
172Yb	CD56	BD	Mouse IgG2b	NCAM16.2		172Yb	CD56	BD	Mouse IgG2b	NCAM16.2
173Yb	H4	Abcam	Mouse IgG1	ab31830		173Yb	H4	Abcam	Mouse IgG1	ab31830
174Yb	H3K27ac	Active Motif	Mouse IgG1	MABI 0309		174Yb	CENP-A	MBL	Mouse IgG1	3-19
175Lu	CD19	BioLegend	Mouse IgG1	HIB19		175Lu	CD19	BioLegend	Mouse IgG1	HIB19
176Yb	HLA-DR	BioLegend	Mouse IgG2a	L243		176Yb	HLA-DR	BioLegend	Mouse IgG2a	L243
209Bi	CD16	Fluidigm	Mouse IgG	3G8		209Bi	CD16	Fluidigm	Mouse IgG	3G8

Table S2. Clinical and genetic characteristics of JMML patients cohort.

Sample ID No.	MDS ID	Age (years)	Tissue	Genotype	Karyotype	Interval diagnosis to splenectomy [days]	Clinical outcome
#6	D 123	2.2	Spleen	PTPN11	normal	69	RRM
#7	D 124	0.5	Spleen	PTPN11	normal	34	RRM
#12	D 217	1.2	Spleen	PTPN11	normal	77	Remission (FUP-16years)
#15	I 187	1.9	Spleen	PTPN11	normal	632	Remission (FUP-12 years)
#16	I 214	2.5	Spleen	PTPN11	Mo7	126	TRM

FUP: follow up; TRM: Transplant related mortality; RRM: Relapse-related mortality.

Note: Splenectomies were performed prior to hematopoietic stem cell transplantation in all 5 cases. Leukemia burden was high in all 5 patients at the time of splenectomy.

Table S3. Phenotype of hematopoietic stem and progenitor subsets identified in UCBs and. JMML patients samples.

S.No.	HSPC subset	Phenotype
1	Hematopoietic stem cells (HSCs)	$Lin^-CD34^+CD38^-CD45RA^-CD90^+$
2	Multipotent progenitor (MPP)	$Lin^-CD34^+CD38^-CD45RA^-CD90^-$
3	Lympho-myeloid primed progenitor cell (LMPP)	$Lin^-CD34^+CD38^-CD45RA^+CD90^-$
4	Common myeloid progenitor (CMP)	$Lin^-CD34^+CD38^+CD123^+CD45RA^-$
5	Granulocyte-monocyte progenitor (GMP)	$Lin^-CD34^+CD38^+CD123^+CD45RA^+$
6	Plasmacytoid dendritic cells (pDC)	$Lin^-CD34^+CD38^+CD123^+CD45RA^{High}$
7	Leukemic-multipotent-like progenitor (L-MPP)	$Lin^-CD34^+CD38^-CD90^+CD45RA^+$
8	Progenitor-like (HPC) subset	$Lin^-CD34^+CD38^+CD90^+CD45RA^+$

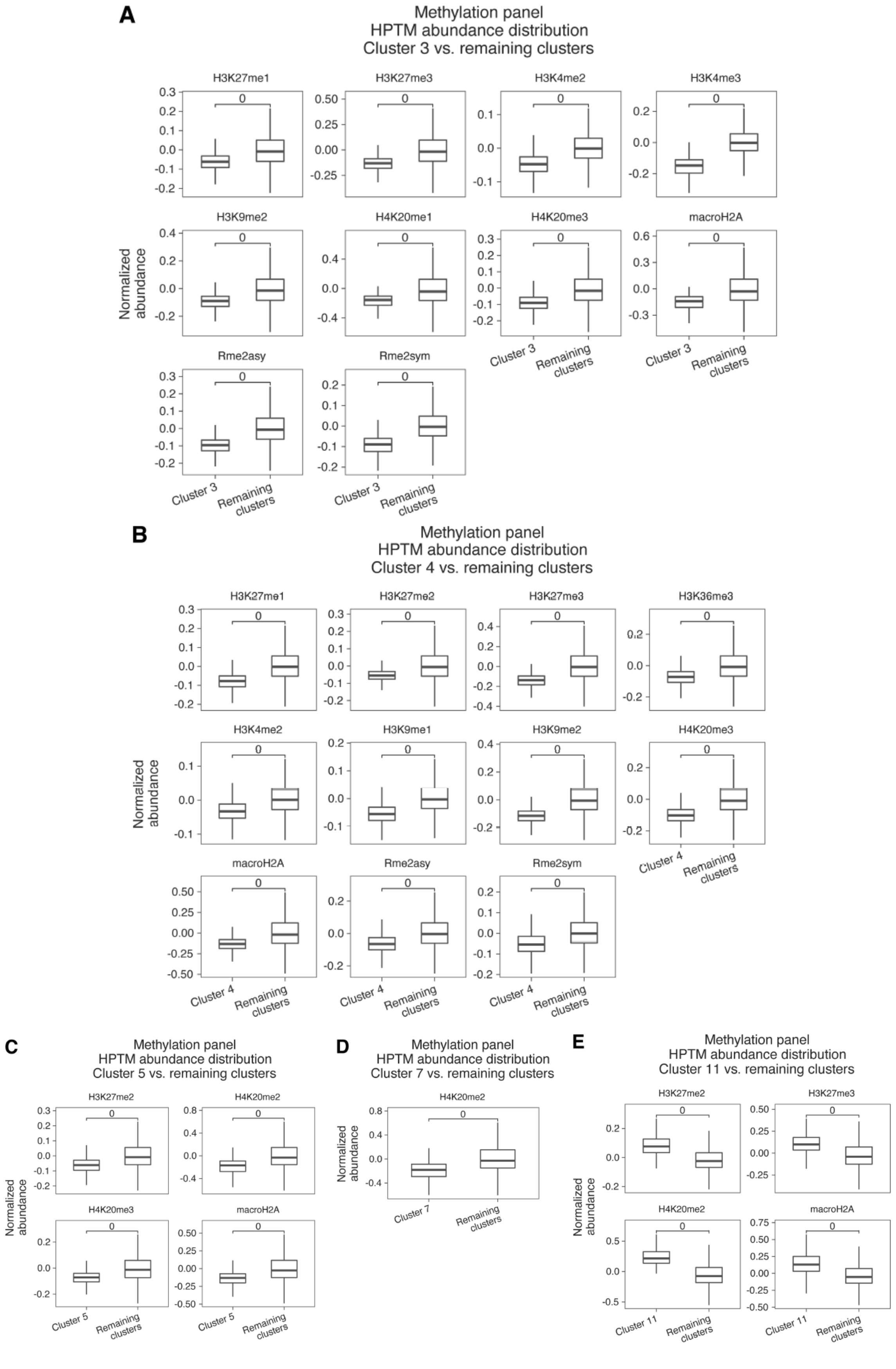

Figure S1: Key histone methylation marks that defined clusters with significantly distinct JMML vs UCB HSPC proportions: Dimensionality reduction analysis was performed on normalized datasets using uniform manifold approximation and projection (UMAP) based on histone methylation post translational modification (PTM) marks. Thereafter, clustering was done using PhenoGraph which

revealed 13 distinct clusters of which clusters A) 3, B) 4, C) 5, D) 7 and E) 11 showed significantly distinct distribution of JMML vs UCB HSPCs. We examined median abundance of HPTMs in each of these individual clusters against all the other clusters to identify the HPTM marks that were significantly distinct (higher or lower) abundant in each cluster. Statistical significance was calculated using t-tests and p values in all cases shown here are <0.0005.

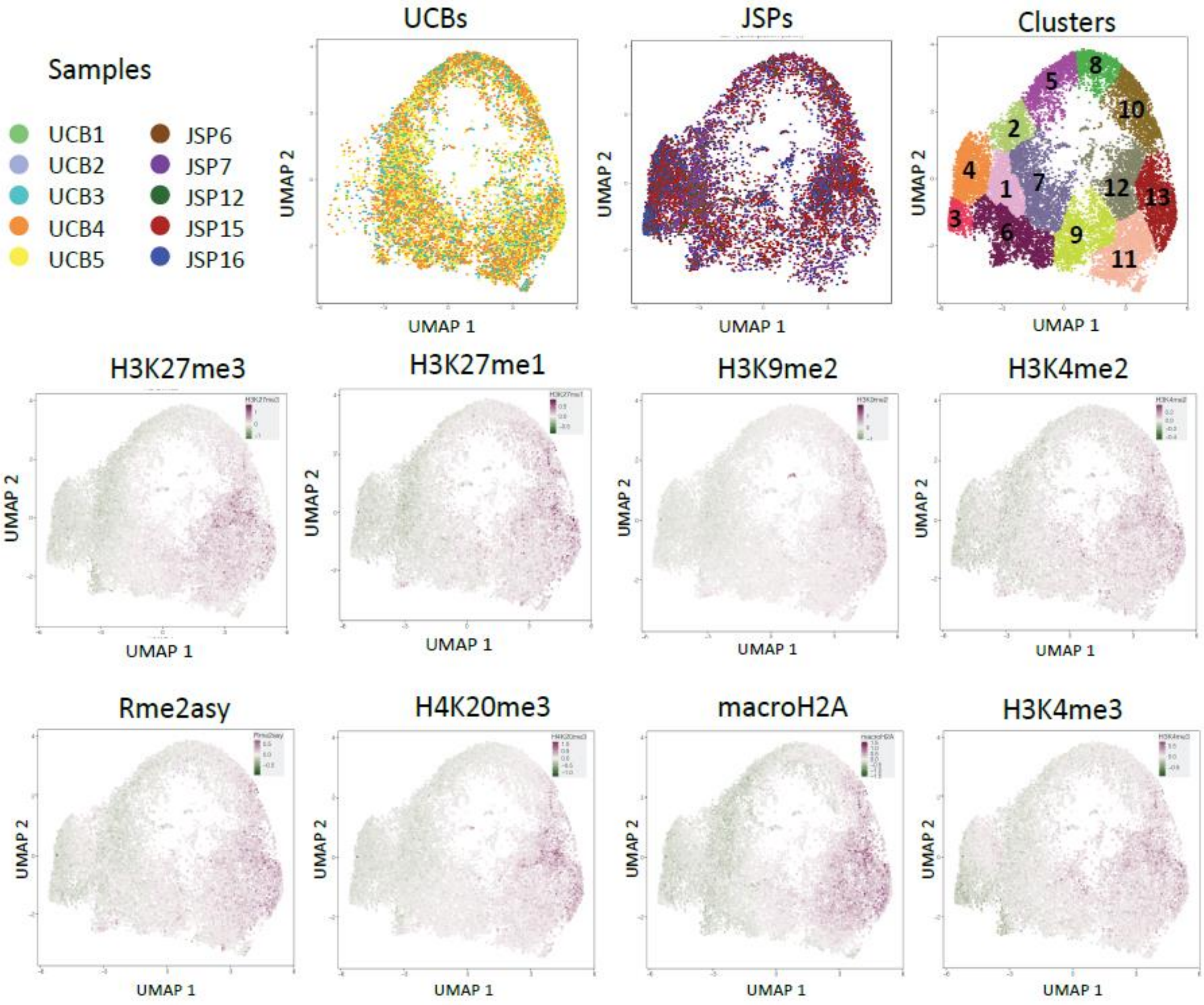

Figure S2: Distinct histone methylation signatures in JMML versus UCB HSPCs using UMAP clustering. Dimensionality reduction analysis was performed on normalized datasets using uniform manifold approximation and projection (UMAP) based on histone methylation post translational modification (PTM) marks in Table S1. Individual contour UMAP plots of all UCBs or all JMML spleens are also shown alongside the clustering map wherein 13 distinct clusters were identified with varied distribution of JMML spleens (n=5) and UCBs (n=5) cells within each cluster. Significant loss of H3K27me3, H3K27me1, H3K9me2, H3K4me2, Rme2asy, H4K20me3, macroH2A and H3K4me3 was observed in JMML SP HSPCs when compared to UCBs. Color represents median histone PTM expression as indicated from minimum (green) to maximum (magenta) in each UMAP plot. HSPC: hematopoietic stem or progenitor cells; UCB: umbilical cord blood; SP: Spleen; UMAP: uniform manifold approximation and projection.

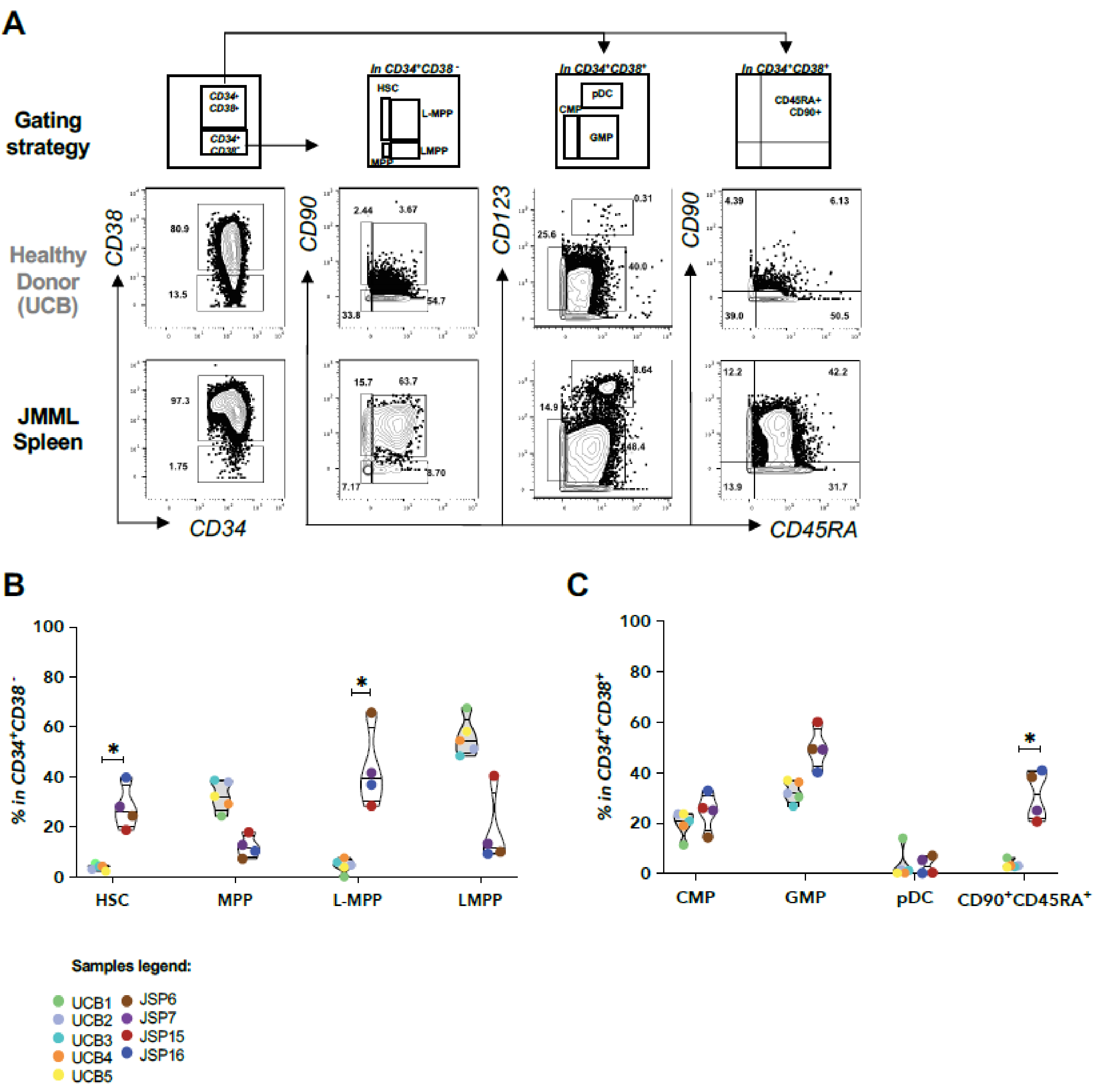

Figure S3: HSPCs identified in JMML samples compared to healthy donor controls. HSPCs distribution in healthy donor controls (UCB n=5) and JMML patient samples at diagnosis (SP n=4) using EpiTOF are shown. A) Gating strategy implemented for distinct HSPC subsets in control and JMML groups. Quantifications of the classical and novel HSPCs identified include B) HSC, MPP, leukemic MPP (L-MPP), LMPP, C) GMP, CMP, pDC and CD34+CD38+CD90+CD45RA+, shown in violin-jitter plots as Mean+SD. Sample legends of HD UCBs and JMML spleens are color coded consistent with figures 1 and 2. Multiple t-tests were performed for statistical analysis (* : $p<0.05$, ** :$p<0.01$, *** : $p<0.001$). EpiTOF: Epigenetic landscape profiling using cytometry by Time-Of-Flight; HSPC: Hematopoietic stem or progenitor cells; UCB: umbilical cord blood; SP: Spleen; BM: Bone marrow; HSC: Hematopoietic stem cells; MPP: multipotent progenitor; LMPP: Lymphoid-myeloid primed progenitor; L-MPP: Leukemia-multipotent-like progenitor; pDC: Plasmacytoid dendritic cells; GMP: Granulocyte-macrophage progenitor; CMP: Common myeloid progenitor.

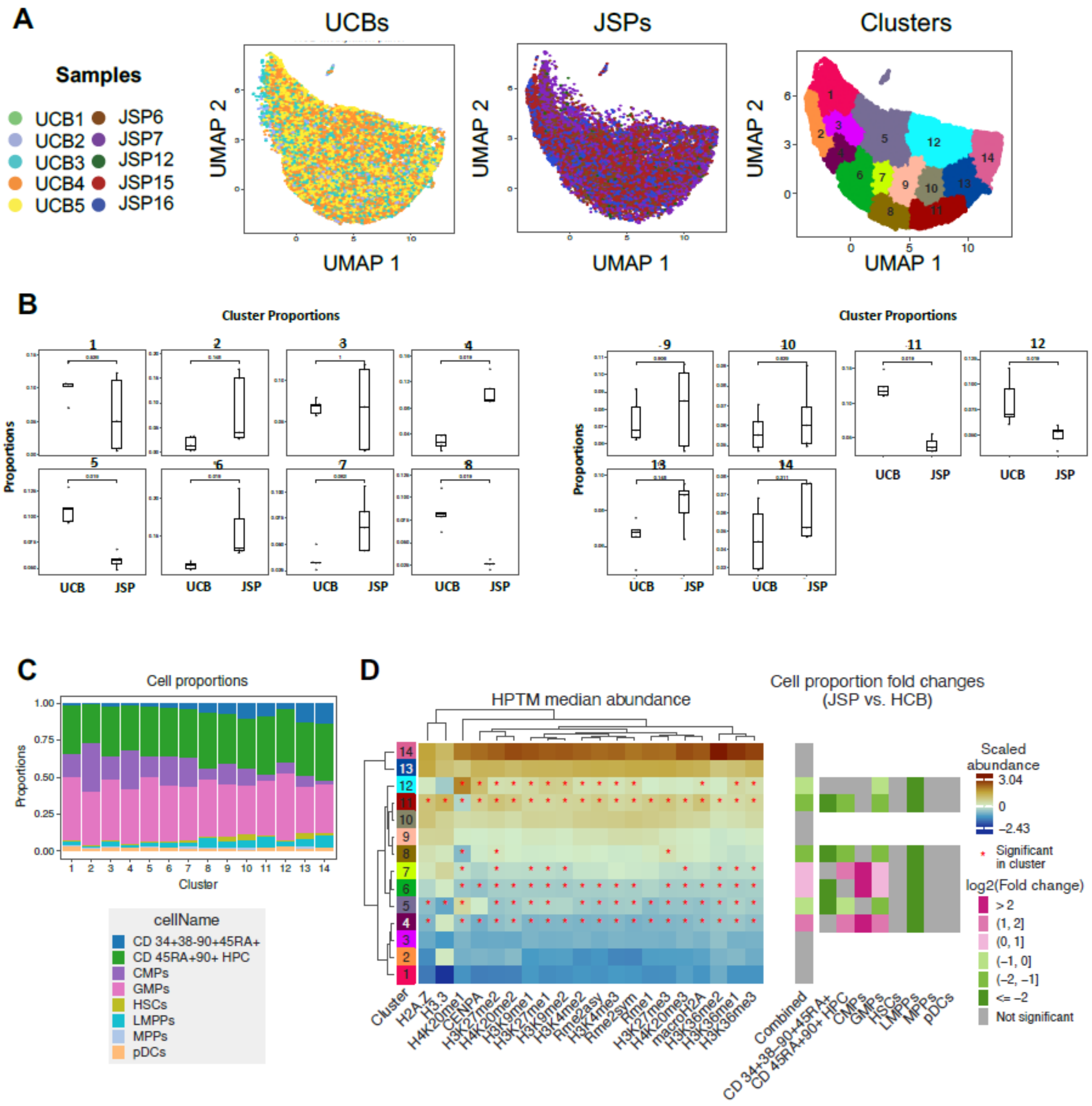

Figure S4: Reduction in histone methylation marks in splenic JMML HSPC subsets**.** A) Dimensionality reduction analysis was performed on normalized datasets of isolated individual HSPC subsets (Table S3) from each JMML patient spleen samples and control UCBs using uniform manifold approximation and projection (UMAP) based on histone methylation post translational modification (PTM) marks in Table S1. Single-cell level UMAP of HSPCs from UCBs (n=5) or JMML SPs (n=5) are generated with each dot representing a single HSPC cell, and each sample is color coded as per the legend. Clustering map is also shown displaying the 14 distinct clusters that were identified with varied distribution of JMML spleens (n=5) and UCBs (n=5) HSPC subsets within each cluster. B) Clusters 4,6 and 7 have significantly higher proportion of HSPCs from JMML samples. Clusters 5, 8, 11 and 12 have significantly higher proportion of UCB HSPCs. Each dot represents a single sample, and each patient sample is color coded as per the sample legend. C) Each of the 14 clusters had heterogenous distribution of UCB and JMML HSPCs as shown in the stacked bar plots with the legend denoted below the graph. D) Heatmaps were generated for visualization of median abundance of histone methylation marks per UMAP-cluster based on unsupervised hierarchical clustering. Significant abundance or loss of the histone PTM marks that define each cluster distinctly from all the other clusters are marked by an *. The fold change in JMML vs UCB cell proportion per cluster is also highlighted, with JMML-abundant clusters (4, 6 and 7) being marked magenta and UCBabundant clusters (5,8, 11 and 12) marked green. Each cluster is defined by distinct histone PTMs signature. Overall differences in histones PTM marks profiles between clusters as well as between individual histone PTMs, are denoted with dendrograms.

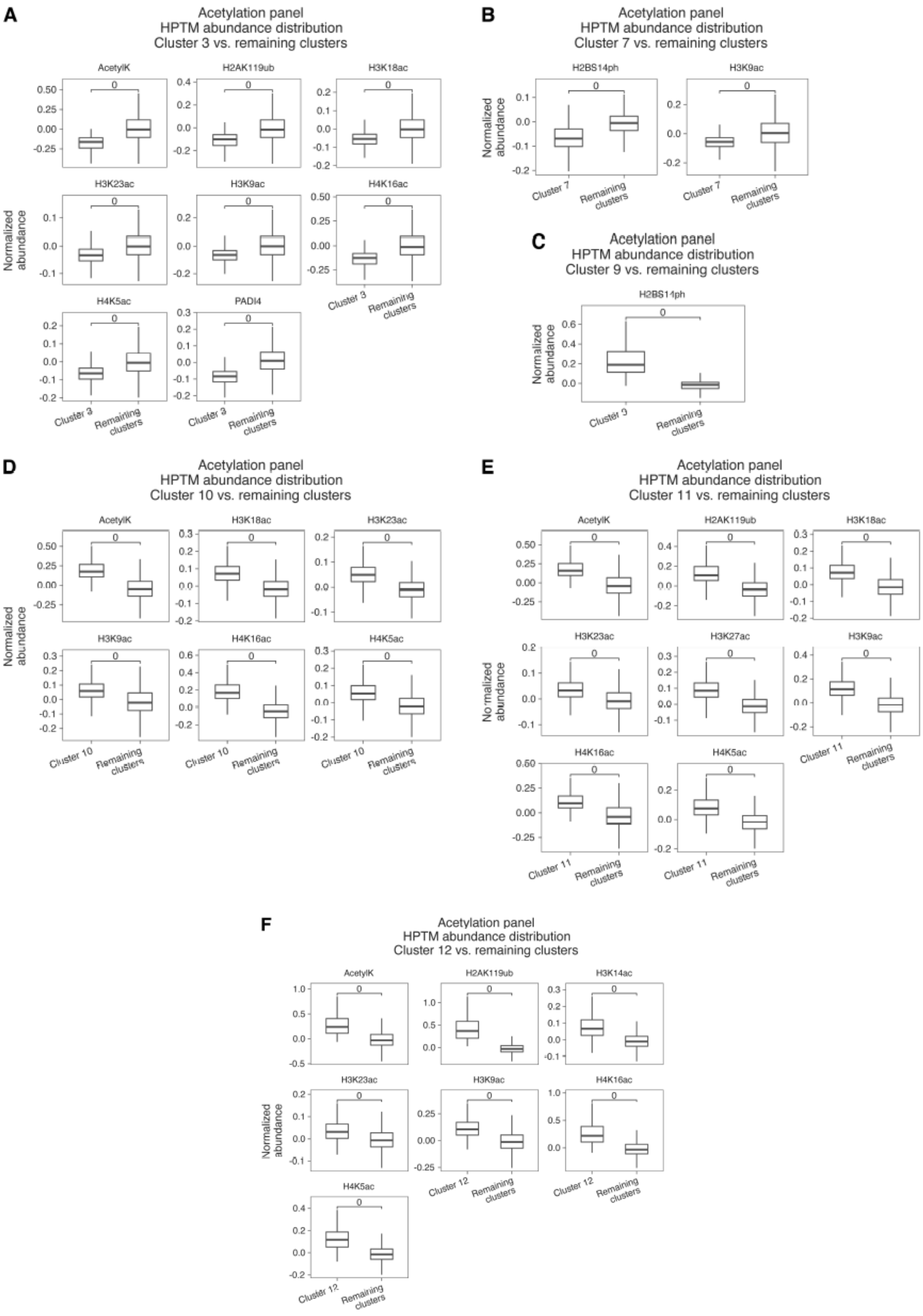

Figure S5: Key histone acetylation, phosphorylation, and ubiquitination marks that defined clusters with significantly distinct JMML vs UCB HSPC proportions: Dimensionality reduction analysis was performed on normalized datasets using uniform manifold approximation and projection (UMAP) based on histone acetylation, phosphorylation, and ubiquitination post translational modification (PTM) marks. Thereafter, clustering was done using PhenoGraph which revealed 12 distinct clusters of which clusters A) 3, 5, B) 7, C) 9, D) 10, E) 11 and F) 12 showed significantly distinct distribution of JMML vs UCB HSPCs. We examined median abundance of HPTMs in each of these individual clusters against all the other clusters to identify the HPTM marks that were significantly distinct

(higher or lower) abundant in each cluster. Statistical significance was calculated using t-tests and p values in all cases shown here are <0.0005.

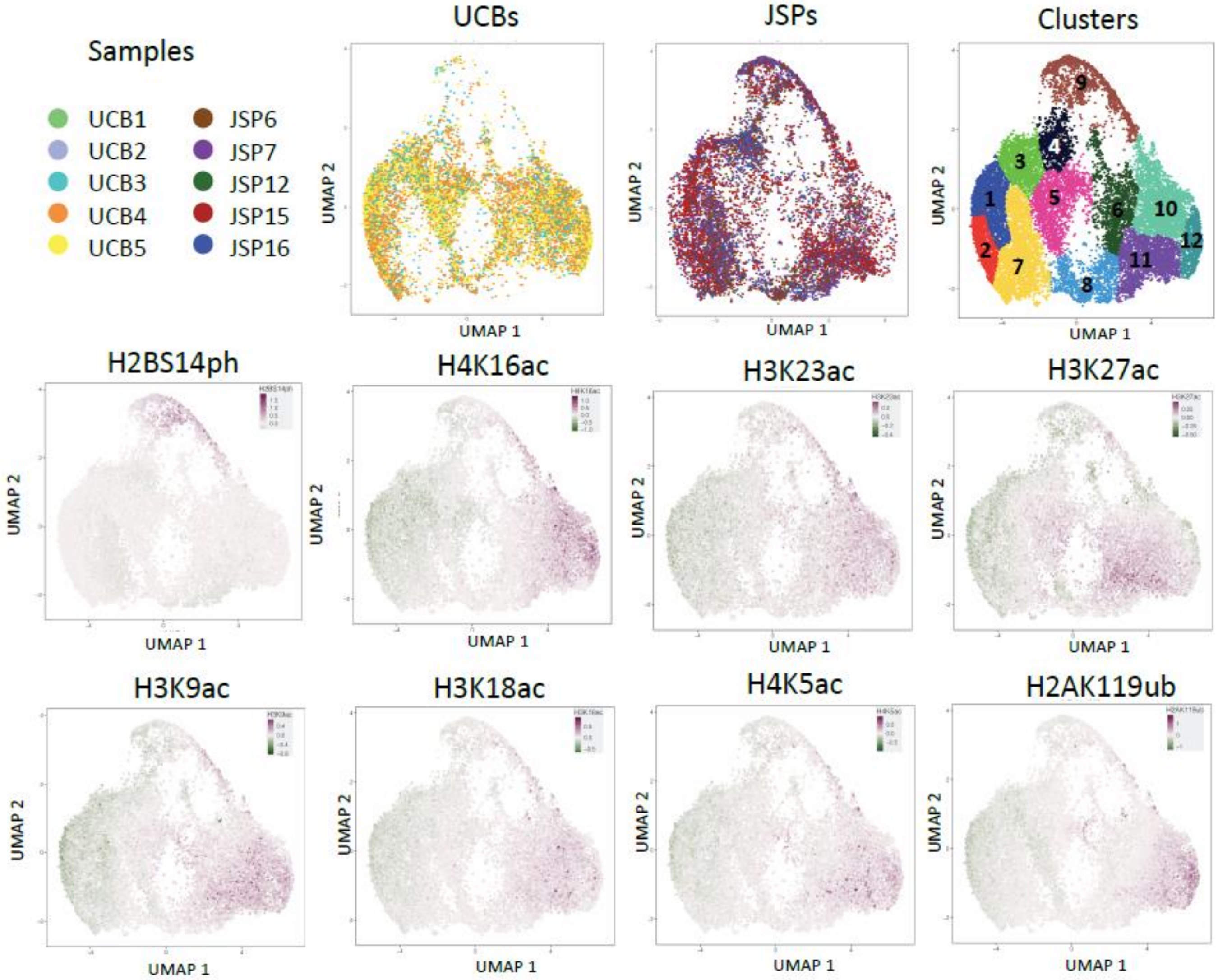

Figure S6: Distinct histone acetylation, phosphorylation, and ubiquitination signatures in JMML versus UCB HSPCs using UMAP clustering. Dimensionality reduction analysis was performed on normalized datasets using uniform manifold approximation and projection (UMAP) based on histone acetylation, phosphorylation, and ubiquitination post translational modification (PTM) marks in Table S1. Individual contour UMAP plots of all UCBs or all JMML spleens are also shown alongside the clustering map wherein 12 distinct clusters that were identified with varied distribution of JMML spleens (n=5) and UCBs (n=5) cells within each cluster. Overall trend of reduced H3K9ac, H4K16ac, H3K23ac, H3K27ac, H4K5ac, and H2AK119ub was observed in JMML SP HSPCs when compared to UCBs. Varied expression of H2BS14ph mark was observed within JMML-abundant clusters 7 and 9. Color represents histone marker expression as indicated from minimum (green) to maximum (magenta) in each UMAP plot. HSPC: hematopoietic stem or progenitor cells; UCB: umbilical cord blood; SP: Spleen; UMAP: uniform manifold approximation and projection.

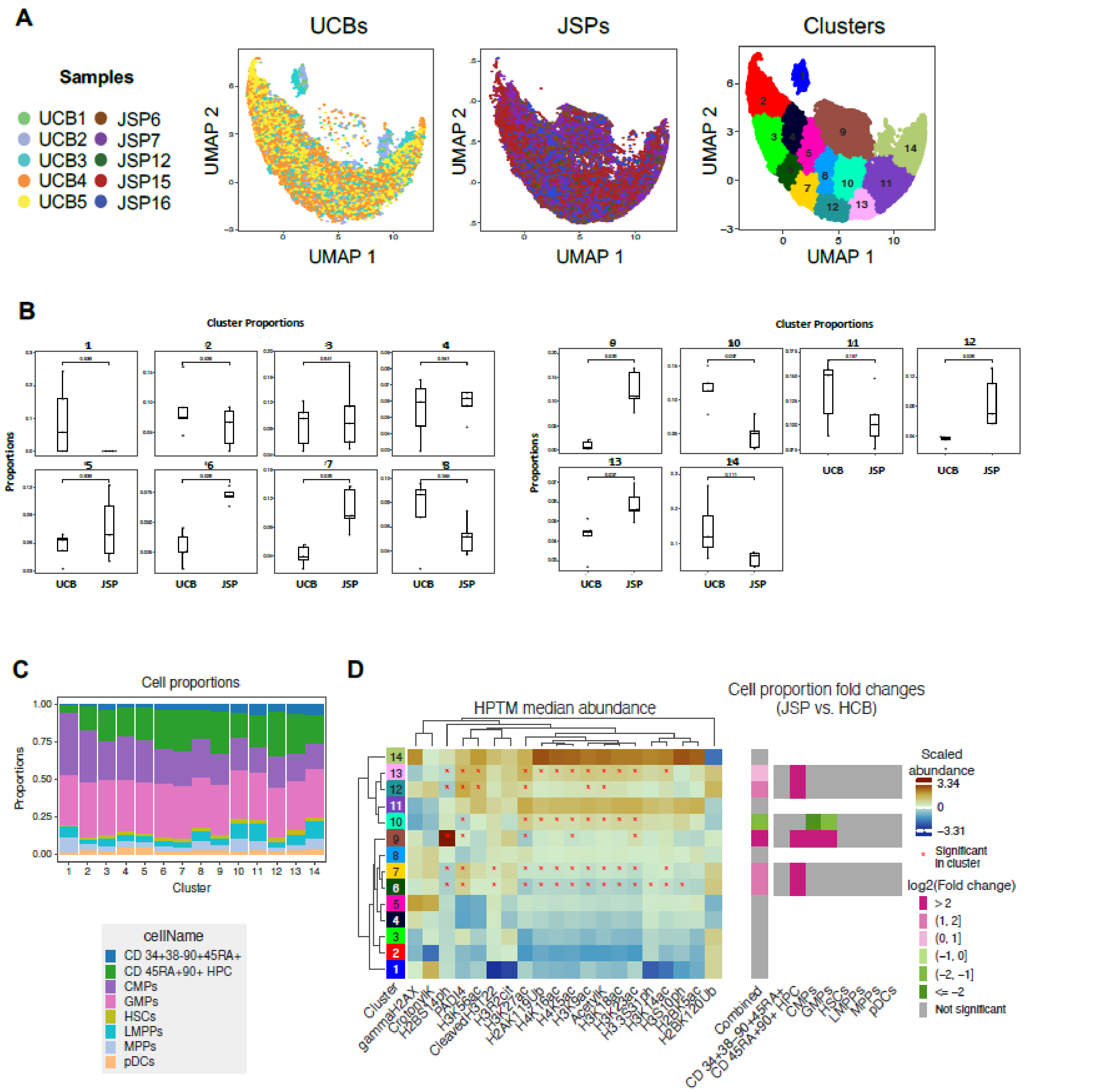

Figure S7: Heterogenous histone acetylation, ubiquitination and phosphorylation markers in primary JMML splenic-HSPC subsets. A) Dimensionality reduction analysis was performed on normalized datasets of isolated individual HSPC subsets (Table S3) from each JMML patient spleen samples and control UCBs using uniform manifold approximation and projection (UMAP) based on histone acetylation, ubiquitination and phosphorylation post translational modification (PTM) marks in Table S1. Single-cell level UMAP of HSPCs from UCBs (n=5) or JMML SPs (n=5) are generated with each dot representing a single HSPC cell, and each sample is color coded as per the legend. Clustering map is also shown displaying the 14 distinct clusters that were identified with varied distribution of JMML spleens (n=5) and UCBs (n=5) HSPC subsets within each cluster. B) Clusters 6, 7, 9, 12 and 13 have significantly higher proportion of HSPCs from JMML samples. While only cluster 10 has significantly higher proportion of UCB HSPCs. Each dot represents a single sample, and each patient sample is color coded as per the sample legend. C) Each of the 14 clusters had heterogenous distribution of UCB and JMML HSPCs as shown in the stacked bar plots with the legend denoted below the graph. D) Heatmaps were generated for visualization of median abundance of histone methylation marks per UMAP-cluster based on unsupervised hierarchical clustering. Significant abundance or loss of the histone PTM marks that define each cluster distinctly from all the other clusters are marked by an *. The fold change in JMML vs UCB cell proportion per cluster

is also highlighted, with JMML-abundant clusters (6, 7, 9, 12 and 13) being marked magenta and UCBabundant cluster 10 marked green. Each cluster is defined by distinct histone PTMs signature. Overall differences in histones PTM marks profiles between clusters as well as between individual histone PTMs, are denoted with dendrograms.
